# Pharmacotechnical Development of a Nasal Drug Delivery Composite Nanosystem Intended for Alzheimer’s Disease Treatment

**DOI:** 10.3390/pharmaceutics12030251

**Published:** 2020-03-11

**Authors:** Thomas Adnet, Anne-Claire Groo, Céline Picard, Audrey Davis, Sophie Corvaisier, Marc Since, Frédéric Bounoure, Christophe Rochais, Loïc Le Pluart, Patrick Dallemagne, Aurélie Malzert-Fréon

**Affiliations:** 1 Normandie Univ, UNICAEN, CERMN, 14000 Caen, France; adnet.thomas.ph@gmail.com (T.A.); audrey.davis@unicaen.fr (A.D.); sophie.corvaisier@unicaen.fr (S.C.); marc.since@unicaen.fr (M.S.); christophe.rochais@unicaen.fr (C.R.); patrick.dallemagne@unicaen.fr (P.D.); 2 CHU, 14000 Caen, France; 3 UNILEHAVRE, FR 3038 CNRS, URCOM, EA 3221, Normandie University,76063 Le Havre, France; celine.picard@univ-lehavre.fr; 4 UFR of Health, Laboratory of Pharmaceutical & Biopharmaceutical technology, UNIROUEN, Normandy University, 76183 Rouen CEDEX, France; frederic.bounoure@univ-rouen.fr; 5 LCMT, UMR CNRS 6507, EnsiCaen UniCaen, 14000 Caen, France; loic.lepluart@ensicaen.fr

**Keywords:** nose-to-brain delivery, nanocomposite, thermosensitive hydrogel

## Abstract

Direct nose-to-brain delivery has been raised as a non-invasive powerful strategy to deliver drugs to the brain bypassing the blood-brain barrier (BBB). This study aimed at preparing and characterizing an innovative composite formulation, associating the liposome and hydrogel approaches, suitable for intranasal administration. Thermosensitive gel formulations were obtained based on a mixture of two hydrophilic polymers (Poloxamer 407, P407 and Poloxamer 188, P188) for a controlled delivery through nasal route via liposomes of an active pharmaceutical ingredient (API) of potential interest for Alzheimer’s disease. The osmolarity and the gelation temperature (T° sol-gel) of formulations, defined in a ternary diagram, were investigated by rheometry and visual determination. Regarding the issue of assays, a mixture composed of P407/P188 (15/1%, *w/w*) was selected for intranasal administration in terms of T° sol-gel and for the compatibility with the olfactory mucosal (280 ± 20 mOsmol, pH 6). Liposomes of API were prepared by the thin film hydration method. Mucoadhesion studies were performed by using mucin disc, and they showed the good natural mucoadhesive characteristics of in situ gel formulations, which increased when liposomes were added. The study demonstrated successful pharmacotechnical development of a promising API-loaded liposomes in a thermosensitive hydrogel intended for nasal Alzheimer’s disease treatment.

## 1. Introduction

Alzheimer’s disease (AD) is the most frequent cause of dementia among the elderly [1]. This disease, characterized by an insidious decline in cognitive and non-cognitive functions, is devastating for patients, their family and society. Many types of neurotransmitters are affected in this chronic and progressive neurodegenerative disorder, and the relative importance of each in relation to clinical findings has not been fully elucidated. Today, no curative treatment exists. While current clinical therapy for AD patients is mainly symptomatic treatment enhancing cholinergic function using inhibitors of acetylcholinesterase (AChE) [2], the development of new drugs for a disease modifying approach is essential. Butyrylcholinesterase (BuChE) is a serine hydrolase related to acetylcholinesterase that catalyzes the hydrolysis of choline and non-choline esters, including acetylcholine [3]. Consideration of the neurobiology of BuChE is particularly relevant nowadays in the treatment of neurodegenerative disorders, such as AD. Selective inhibitors of BuChE have been proposed for their potential to treat Alzheimer’s disease [4].

AD and other types of neurodegenerative diseases are difficult to treat in particular because of the blood-brain barrier (BBB). As BBB restricts transport of drugs to the brain, very few drugs are actually available for treatment of AD [5]. While preventing the neurotoxic xenobiotics’ access into the central nervous system (CNS) to protect it, it also strongly limits the administration of neuroprotective drugs to the CNS. The direct nose-to-brain delivery has appeared as a non-invasive powerful strategy to deliver drugs to the brain bypassing the BBB [6]. The most important direct pathway to the brain is the transport or the diffusion directly to the brain through the olfactory mucosa pathway in the olfactory region [7].

The intranasal delivery enhances targeting and reduced systemic side effects [8]. The direct nose-to-brain transport can reduce drug distribution to non-targeted sites, minimizing adverse effects. Scientists started to look for different approaches for brain delivery of drugs, and nasal administration has recently gained special interest. There are various approaches to facilitate nose-to-brain drug delivery, and among them, one finds the use of gelling formulation that inhibits the mucociliary clearance, and that of drug delivery nanosystems [9]. We chose to combine both strategies by the development of a nasal drug delivery composite nanosystem to deliver our active pharmaceutical ingredient (API) in the brain. This formulation is a thermogelling system, composed of liposomes. Firstly, these carriers may provide favorable characteristics to the drug such as enhanced absorption, mucoadhesion and increased stability. Secondly, using gels increases drug residence time in the nasal cavity for a better absorption.

Various API loaded in nanoparticles and intranasally administered were studied for the treatment of AD [10,11]. For example, the intranasal administration of a donepezil-loaded liposome significantly increased drug delivery to the brain compared with the conventionally used products [12]. Similarly, the intranasal administration of rivastigmine-loaded liposomes enhanced brain concentrations of rivastigmine compared to the free drug [13]. These results were in accordance with other studies based on rivastigmine liposomal formulations [14,15], indicating the suitability of this approach in the treatment of AD.

As our API presents a high aqueous solubility (> 10 mg/mL) and a limited permeability, it could be considered as a class III drug according to the drug biopharmaceutics classification system (BCS) [16]. Hydrophilic API are not suitable for absorption through nasal mucosa. As a consequence, loading the drug into flexible liposome overcomes this obstacle and facilitates the drug transfer across mucosa [17].

Liposomes can encapsulate hydrophilic and hydrophobic drugs. Hydrophobic drugs have affinity to the phospholipid bilayer, while hydrophilic drugs are entrapped in the aqueous cavity [18]. Liposomes are biocompatible, completely biodegradable, safe and non-immunogenic [19]. Thus, encapsulation into their aqueous core appears as a promising strategy for the brain delivery of hydrophilic API by intranasal route. 

To optimize API remanence, we have chosen to develop an in situ forming gel: a polymer solution which gelifies in response to an external stimulus [20], here, the temperature. Being thermoresponsive, the system is intended to behave as a solution at room temperature in order to facilitate administration, and to become a gel in the nasal cavity at 34 °C. 

Poloxamer is a water-soluble and, non-ionic triblock copolymer. It is constituted of a hydrophobic polyoxypropylene (POP) central block and two hydrophilic blocks of polyoxyethylene (POE). There are many types of Poloxamers, which differ in their molecular weight and the ratio of the POP and POE units present. Among the most frequently encountered Poloxamers are Poloxamers 188 (P188, POE_75_-POP_30_-POE_75_) and 407 (P407, POE_101_-POP_56_–POE_101_). Due to their reversible thermal characteristics, Poloxamer-based hydrogels are interesting candidates as drug carriers. Consequently, these thermoreversible and not irritating gel have been largely investigated to develop mucoadhesive formulations [21]. P188 is interesting to use in association with P407 in order to modulate the Tsol-gel and the properties of gel. Indeed, Soliman et al. showed that P188 increased the Tsol-gel and mucoadhesive force of in situ thermo-sensitive gels for the vaginal administration of sildenafil [22]. Recently, Poloxamer 407 was used alone or with mucoadhesive polymer to prepare in situ hydrogels for therapy of neurodegenerative disease including Alzheimer’s disease [23,24].

The *in situ* gel system containing liposomes enables mucoadhesion, improvement of drug permeability, and a sustained and controlled delivery [25,26].

To optimize the persistence of the gel in the intranasal cavity, it may be advantageous to use mucoadhesive formulations by incorporating other polymers into the gel formulation. Thus, the addition of well know mucoadhesive polymers such as chitosan or hydroxypropyl methyl cellulose (HPMC) would improve mucoadhesive properties of the developed formulation.

Intranasal administration has been privileged because it is a non-invasive, painless method and usable for ambulatory treatment [27]. 

Hence, the aim of this study is to develop and characterize an innovative composite formulation, associating the liposome and hydrogel approaches, suitable for intranasal administration. To avoid adversely affecting the functions of the nasal mucosa and its cilia, we aimed that this formulation corresponds to European pharmacopeia criteria about nasal preparations, i.e., to be an isotonic formulation (280 ± 20 mOsmol), with a stabilized pH value (6 ± 3) [28].

## 2. Materials and Methods 

### 2.1. Materials (AD)

Soybean, and egg phosphatidylcholine (SPC and EPC, respectively) were gifts from Lipoid GmbH (Ludwigshafen, Germany). Cholesterol (CHOL), Sepharose^®^ CL-4B, and Chitosan oligosacharide lactate, donepezil, hydroxypropylmethyl cellulose, gellan gum, acetylthiocholine-iodide (ATC), butyrylthiocholine iodide and 5,5-dithiobis-(2-nitrobenzoic) acid (DTNB), human aceylcholinesterase (AChE), equine butyrylcholinesterase (BuChE), donepezil were purchased from Sigma-Aldrich (Ludwigshafen, Germany). Tacrine was provided from Tocris (Lille, France). Kolliphor® P407 (POE_101_-POP_56_-POE_101_ triblock copolymer) and Kolliphor® P188 (POE_75_-POP_30_-POE_75_ triblock copolymer) were gifts from BASF (Levallois-Perret, France). Sodium chloride was purchased from Carlo Erba (Val de Reuil, France). Prisma Buffer HT pH 7,4, sink brain buffer, corticosterone and theophylline was obtained from pION (Billerica, MA, Etats-Unis, USA). Methanol, acetonitrile, water of HPLC grade, formic acid, sodium hydroxide, and hydrochloric acid were provided by Prolabo VWR International (Fontenay-sous-Bois, France).

### 2.2. Characterization of the Active Pharmaceutical Ingredient

#### 2.2.1. In Vitro Measurement of AChE and BuChE Activity 

The API inhibitory capacity on acetylcholinesterase (AChE) and Butyrylcholinesterase (BuChE) biological activity was evaluated through the use of the spectrometric method of Ellman [29], and realized according to the protocol already reported by our team [30]. Donepezil or tacrine were used as a reference standard. Assays were performed with a blank containing all components except acetyl- or butyrylthiocholine, to account for non-enzymatic reactions. The percent inhibition due to the presence of the test compound was calculated by the following expression: ((v_0_–v_i_)/v_0_) × 100, where v_i_ is the rate calculated in the presence of inhibitor and v_0_ is the enzyme activity. 

IC_50_ values were determined graphically by plotting the % inhibition versus the logarithm of six inhibitor concentrations in the assay solution, using the GraphPad Prism software (version 6.01, GraphPad Software, La Jolla, CA, USA). All experiments were performed in *n* = 3.

#### 2.2.2. Parallel Artificial Membrane Permeability Assay (PAMPA)

The PAMPA-GIT experiments were conducted using the Pampa Explorer Kit (Pion Inc., Billerica, MA, USA) according to manufacturer’s protocol, and realized according to the protocol already reported by our team [31]. Quality control standards with known Pe were used as references: the low/moderately permeable corticosterone (Pe = 202.0 nm/s at pH = 7.4) and theophylline (Pe = 5.0 nm/s at pH = 7.4).

### 2.3. Preparation of Liposomes

#### 2.3.1. Formulation of Liposomes

Liposomes were formulated according to the adapted method of the thin lipid film hydration [32]. A lipid blend of soybean phosphatidylcholine (SPC) and cholesterol (CHOL) was used at a molar ratio 8:1. Lipid solutions in chloroform/methanol (4:1) were evaporated under nitrogen flow, and left under vacuum for 3–4 h to form a lipid film. This thin lipid film was then hydrated in water or API solution, and vortexed. The yielded multilamellar vesicles (MLVs) were then extruded 13 times with a mini-extruder (Avanti Polar Lipids, Inc., Alabaster, AL, USA) through polycarbonate membranes with a pore diameter of 100 nm (Avanti Polar Lipids, Inc., Alabaster, AL, USA) to form large unilamellar vesicle (LUVs).

#### 2.3.2. Characterization of Liposomes by Dynamic Light Scattering

The average hydrodynamic diameter associated with the polydispersity index (PDI) of the formulated LUVs and the formulated LUVs dispersed in polymer solution were measured by dynamic light scattering (DLS) using a NanoZS^®^ apparatus (Malvern Instruments SA, Worcestershire, UK). The zeta potential was calculated from the electrophoretic mobility using the Smoluchowski equation, also using a NanoZS^®^ apparatus. The measurements were performed in triplicate at 25 °C, after a 1:100 dilution in water. 

#### 2.3.3. Entrapment Efficiency

Entrapment of the active pharmaceutical ingredient (API) into the liposome was determined. Liposomes were separated by Sepharose^®^ CL-4B column. The API content in the liposome fraction was analyzed by HPLC and the encapsulation efficiency (% EE) was calculated by considering the initial amount of drug added in the formulation using Equation (1):

(1) $$\%\;\mathrm{EE}=\frac{\mathrm{The}\;\mathrm{amount}\;\mathrm{present}\;\mathrm{in}\;\mathrm{liposomes}\;\mathrm{fraction}\;}{\mathrm{Total}\;\mathrm{amount}\;\mathrm{of}\;\mathrm{API}\;\mathrm{added}}\;\times \;100$$

Drug loading (DL) was calculated using Equation (2):

(2) $$\%\;\mathrm{DL}=\frac{\mathrm{Weight}\;\mathrm{of}\;\mathrm{API}\;\mathrm{entrapped}\;\mathrm{within}\;\mathrm{liposomes}}{\mathrm{Total}\;\mathrm{weight}\;\mathrm{of}\;\mathrm{oil}\;\mathrm{phase}\;\mathrm{of}\;\mathrm{the}\;\mathrm{liposomes}}\;\times \;100$$

Total weight of oil phase of the liposome corresponds to the total amount of excipients (excluding the water phase) and API used for the preparation of the liposomes.

#### 2.3.4. UHPLC

API concentrations were measured by high-performance liquid chromatography (UHPLC). The apparatus was composed by an Agilent binary pump 1290, an autosampler 1290 and a diode array UV detector 1260 (Agilent technologies, Santa Clara, CA, USA). The column used was a reversed phase column C18 (Restek^®^ Ultra, 5 µm, 2.1 × 50 mm, Lisses, France). The injection volume and the run-to-run time were 4 μL and 1.53 min, respectively. The flow rate was 0.8 mL/min and the detection wavelength was 214 nm. The mobile phase was initially composed of a mixture of 3% water containing 0.1% (*v/v*) formic acid (A) and 97% acetonitrile containing 0.1% (*v/v*) formic acid (B). Then, the composition reached 90% of B after 0.8 min, then 60% of B after 1.5 min by applying a linear gradient, maintained for 1 min. 5% of B was then reached at 2.51 min, maintained for 1.5 min and returned to the initial conditions. Linearity was observed in the range from 2.5 to 100 µM with a determination coefficient greater than 0.999. The column was maintained at 25 °C. It was checked that in presence of liposome and gel, no interaction with API is present. 

### 2.4. Preparation of the In Situ-Forming Gel 

#### 2.4.1. Gel Formulation

The gel was made on a weight basis using the “cold method” [33]. Poloxamer P407 was mixed with Poloxamer P188, the API, chitosan, gellan gum, HPMC or liposomes and demineralized water (pH 6–7) under magnetic stirring at 500 rpm during at least 30 min at 4 °C, until a homogenous solution was obtained. After dissolution, solutions were stored at 4 °C for 2 h before characterization. Concentrations of Poloxamer P407 (14–25%, *w/v*) and Poloxamer P188 (0–10%, *w/v*) were optimized to obtain an instantly gelling system at physiological conditions. API concentration of 1 mg/mL was used.

#### 2.4.2. Gel Characterization

##### Physicochemical Properties

The pH of formulation was measured using a pH-meter (Eutech instrument, Landsmeer, The Netherlands). To determine osmolarity, a micro-osmometer autocal type 13 based on the freezing-point method (Roebling, Berlin, Germany) was used. 100 µL of each formulation were introduced in a microtube and measurements were performed. Gelation temperature (T_gel_) of formulations was investigated by visual determination. Briefly, formulation was placed in transparent vials under magnetic stirring s in a water bath heated from temperature of 15 °C to 50 °C (at the rate of 2 °C/min). The gelation temperature was recorded when the magnetic bar stopped moving because an increase in viscosity. The visual determinsation of gelation temperature was confirmed by rheometry method.

##### Rheological Evaluation 

All the rheological characterizations were carried out on a rheometer AR1000 (TA Instruments, Guyancourt, France) equipped with a cone-plate geometry (4 cm, 1.59°). 

Gelation temperature determinations were performed in oscillatory mode. The storage and loss moduli were measured as a function of temperature (from 15 to 50 °C at 5 °C/min) with an oscillatory stress value maintained at 25 Pa in order to ensure being in the linear viscoelastic region in both the liquid and solid state. The frequency was set at 1.0 Hz during the measurements.

The viscosity of the gel forming solutions was measured as a function of shear rate (from 0.1–100 s^−1^ then from 100–0.1 s^−1^) in steady state at 34 °C to evaluate the interactions between the formulation components. 

In order to evaluate the gel strength, storage modulus evolution of hydrogels was recorded during stress sweeps performed above T_gel_ at 50 °C from 0 to 600 Pa in oscillatory mode.

### 2.5. In Vitro Evaluation of Mucoadhesion 

The mucoadhesive strength of empty gels (blank) and those containing liposomes was determined using a Texture Analyzer TA-XT Plus (Stable Micro Systems, Cardiff, UK) and a mucin disc test. Porcine mucin discs (250 mg, 13 mm diameter), prepared by direct compression (10 t for 30 s of pig gastric mucin), were horizontally attached onto the lower face of an inert horizontal polycarbonate probe. A force of 5 g was applied for 120 s to ensure contact between formulations and the mucin disc. Then, the probe was raised at a speed of 0.5 mm/s. The mucoadhesive strength was determined from the force of detachment. Measurements were run at 37 °C. A cylindrical probe P/35 (13 mm diameter, aluminum) was used, and an amount of 5 mL of product was delivered on the base. 

### 2.6. In Vitro Release Profile

Kinetic release studies were performed in nasal simulated fluid at 34 °C using Franz cells. 200 µL of formulations was placed into a cellulose ester dialysis tube with 100 kDa pore size (Spectrum lab, Gardena, CA, USA), and incubated in simulated nasal fluid (SNF) pH 6.5. at 34 °C under magnetic stirring. SNF was an aqueous solution containing 8,77 mg/mL of NaCl, 2.98 mg/mL KCl and 0.59 mg/mL CaCl_2_. The receptor solution was withdrawn (samples of 100 µL), was replaced at various time points by fresh SNF and the amount of API released from the formulations was determined by HPLC after a 10-fold dilution in methanol. All measurements were performed in triplicate.

### 2.7. Statistical Analysis

Results were expressed as mean values ± standard error of the mean (SEM) or as mean values ± standard deviation. A Student’s t-test was used for statistical comparison/analysis, and *P* <0.05 was considered statistically significant.

## 3. Results and Discussion 

### 3.1. Characterization of the Active Pharmaceutical Ingredient (API)

API inhibitor capacity of human AChE and equine BuChE was assessed using the Ellman assay [29]. In this test, Donepezil and Tacrine were used as reference AChE and BuChE inhibitors, respectively. The results are depicted in Table 1. 

API appeared able to inhibit BuChE with IC_50_ value of 573 ± 40 nM, whereas its displays a negligible AChE inhibitor activity with IC_50_ value of 14,520 ± 40 nM. Thus, API shows a specific submicromolar inhibition of BuChE.

Both cholinesterase enzymes AChE and BuChE are involved in the hydrolysis of acetylcholine (ACh). In the healthy brain, AChE predominates on BuChE, that is considered to play a minor role in regulating brain levels of ACh. However, during progression of AD in certain brain regions including cortical areas, AChE activity declines while BuChE activity remains unaffected or progressively increases [34]. Finally, in advanced stages of AD, BuChE is considered to play a prominent role in regulating brain ACh levels [35]. Thus, BuChE appears as a valuable target to improve the cholinergic deficit, due to its increasing role predicted in regulating brain ACh levels as the disease progresses. Furukawa-Hibi et al. demonstrated that BuChE inhibition, as well as AChE inhibition, is a viable therapeutic strategy for cognitive dysfunction in AD. Indeed, a selective BuChE inhibitor provides beneficial effects on the cognitive dysfunction induced by amyloid-β peptide in mice [36]. From our experimental results, our API shows a specific submicromolar inhibition of BuChE without affecting AChE (i.e., 25-fold BuChE selectivity). This activity is very promising, especially if compared with reported equine BuChE inhibitory activity of other compounds, e.g., a new series of pyrazinamides, whose showed IC_50_ micromolar values [37].

The cholinesterase inhibitory activity of new compounds is classically evaluated using equine serum BuChE due to the economic viability of this enzyme. Purified human BuChE has limited availability from commercial sources, making equine BuChE a reasonable alternative [38]. In the study of Wu et al., synthetic compounds showed similar effectiveness in inhibiting BuChE from equine and human plasma, but with a higher potency over the human enzyme [39]. Similarly, Dighe et al. have shown that their lead was 2-fold more potent in inhibiting human BuChE than equine BuChE [40]. In the same way, we could hope for a better activity in human BuChE.

Having a selective inhibitor of BuChE is innovative because there is currently no equivalent drug available on the market. The API could have a positive role in the attention, executive function, emotional memory and behavior of patients at advanced stages AD by inhibiting the BuChE present in CNS neurons.

To determine the capacity to reach the CNS, the permeability must be evaluated. To analyze the passive permeability characteristics of API, we measured BBB permeability by a PAMPA model, well adapted to High Throughput Screening (HTS) [41]. Theophylline (Pe = 5.0 ± 0.0 nm/s), and corticosterone (Pe = 202.0 ± 3.0 nm/s) were used as low and high permeability standards, respectively. The API poorly diffused through the lipid bilayer, since Pe values (≤20.4 ± 3.0 nm/s), significantly lower than the positive control, indicated a potential difficulty in crossing the BBB. Thus, because of this limited permeability and its good water solubility (>10 mg/mL), the API can be considered as a class III BCS drug [16]. This poor BBB permeability, probably related to the hydrophilic character of the API, would lead to a poor brain bioavailability after intravenous administration.

However, whereas intravenous administration of API is unlikely to reach CNS, BuChE inhibition must occur in the CNS to be a viable therapeutic strategy in advanced AD [3]. Moreover, the API peripheral biodistribution could lead to important side effects, if it is not controlled and limited. Indeed, the heart is naturally rich in cholinesterase (ChE). Its inhibition may affect the cardiac function, especially in elderly patients, many of them having concomitant cardiovascular diseases. Peripheral ChE inhibition potentiates the cardio-inhibitory effect with retarding ACh degradation and leads to negative chronotropic and dromotropic effects through muscarinic receptors [42]. As a consequence, a CNS selectivity is highly desired to avoid any dramatic side effect and improve API efficiency. 

Nose-to brain delivery is a powerful strategy to increase this expected CNS selectivity.

Drug nasal delivery enables to bypass BBB and permits the API brain delivery. Nevertheless, this route of administration may be limited by enzyme degradation and quick mucociliary clearance [43] if formulation is not optimized. To increase drug transport efficiency into CNS via intranasal delivery, we have chosen to develop an innovative composite formulation based on liposomes included in gels.

### 3.2. Preparation of Liposomes

Unloaded (i.e., blank) and API-loaded liposomes (API-Lip) were formulated by the thin lipid film hydration method. Unloaded and API-lip were characterized in terms of granulometric properties, zeta-potential, and encapsulation efficiency (Table 2). Various SPC and cholesterol concentrations, and molar ratios were assayed, without any significant influence on the liposomes physico-chemical properties (results not shown).

For the retained liposomal formulation, based on a 120 mM SPC and cholesterol mixture, used in a molar ratio of 8:1, the mean hydrodynamic diameter was 119.0 ± 0.7 nm for blank Lip, and 114.9 ± 2.4 nm for API-Lip. For all the formulations, the polydispersity index (PDI) values were lower than 0.1, indicating monomodal size distributions. Encapsulation efficiency of API in Lip was 11%. This corresponds to an API concentration of 1.2 ± 0.1 mg/mL, and a drug loading of 1.4 ± 0.1%. SPC/cholesterol liposome formulations are widely found in the literature, and the particle size properties obtained are comparable to ours results [44,45].

The nose-to-brain passage of nanoemulsions has been studied through live imaging or ex vivo histological examination in rats after nasal administration by Ahmad et al. [46]. This study demonstrated that nanoparticle size plays a determinant role, by directly influencing their in vivo fate. NEs with a size of about 100 nm have longer retention time in nostrils and slower mucociliary clearance than larger ones (i.e., >200 nm). NEs of 100 nm can be transported through the trigeminal and the olfactory nerves to the olfactory bulb. Subsequent uptake and translocation of nanoparticles along axons of the olfactory nerve have been shown in non-human primates and rodents [47]. Morrison and Costanzo established that the diameter of a human olfactory neuron axon ranges from 100 to 700 nm [48]. Therefore, theoretically, axonal transport of up to ~500 nm diameter particles is possible in humans. Al Asmari et al. showed that liposomal formulation of donepezil with a size of 120 nm, significantly increased brain concentration after intranasal administration [12]. Similarly, pharmacokinetic and brain targeting studies in rats revealed a significantly high concentration of drug in brain following intranasal administration of solid lipid nanoparticles of donepezil in comparison with a donepezil solution [49]. This demonstrates brain targeting efficiency of solid lipid nanoparticles of 120 nm. As a consequence, liposomes of 120 nm appear really suitable for brain targeting through nasal route and could reach the olfactory bulb through axons.

Furthermore, in ours assays, zeta potential values were only very slightly negative, and broadly neutral for both blank Lip and API-Lip. Surface charge of the nanoparticles could influence the mechanisms involved in their transport from the nasal cavity to the CNS. Indeed, the olfactory pathway is mainly responsible for the translocation of negatively charged nanoparticles, whereas the positively charged nanoparticles reach the brain more slowly, by involving the trigeminal pathway [50]. Intranasally applied nanoparticles have to penetrate the mucus layer covering nasal mucosa to reach the olfactory mucosa, and thus the CNS [51]. Interactions between particles and mucus could lead to trapping and wrapping of nanoparticles in mucus, making them less suitable as efficient vehicles for nose-to-brain delivery [27]. Uncharged nanoparticles, which do not interact with mucus are able to diffuse through mucus more efficiently [52]. 

Thus, nanoparticles appear well designed to permeate mucus by being small enough, neutral in net surface charge and coated densely with charged groups that prevent hydrophobic bonding to mucins [53].

The liposomes, that we have formulated, present surface charges close to neutrality and very slightly negative, as well in the absence (potential ζ = - 5.5 ± 0.3 mV) as in the presence of the encapsulated API (potential ζ = - 11.2 ± 1.4 mV), and although in gels (potential ζ = -6.6 ± 0.5 mV) (see after). In view of these particle size and surface charge properties of the formulated liposomes, the prerequisites for allowing the addressing and diffusion of the API towards the olfactory mucosa appear to be satisfied.

### 3.3. Preparation of in Situ-Forming Gel 

We attempted to develop an aqueous formulation of API that allows an increased residence time in the nasal cavity. Hydrogels are a particularly attractive type for intranasal API administration. Widely used in various medical fields [54,55,56], the hydrogels are devoid of potentially toxic organic solvents since they are composed of a large amount of water and a network of cross-linked polymers.

Poloxamers are biodegradable by erosion [57] and biocompatible polymers [58]. Based on these safety data and the information that the manufacturing process can be controlled to limit unwanted impurities, the Cosmetic Ingredient Review Expert Panel concluded that poloxamers are safe as used [59].

Poloxamers are composed of a hydrophobic POP block, sandwiched between two hydrophilic POE blocks [60]. Among them, P407 is a non-ionic surfactant, endowed with gelation properties above a defined sol-gel transition temperature (T_gel_). Below this temperature, the sample remains fluid, and above, the solution becomes semi-solid. This phenomenon of thermogelling is perfectly reversible. When the temperature increases, copolymer macromolecules aggregate into micelles. This micellization is due to the dehydration of hydrophobic POP blocks, which represents the very first step towards gelation. When the polymer concentration is high enough, the micellization is followed by gelation. 

Surprisingly and unlike some empirical data from the literature [61], we experimentally observed that hydrogel formulations based on the sole P407 could not lead to a T_gel_ compatible with our specifications (data not shown). According to information provided by the supplier, the production mode of P407 has recently evolved, thus modifying the average molecular weight of the poloxamers produced, and resulting in a direct modification of the gelation temperatures. Hence, to further modulate sol-gel temperature of P407 hydrogel, P188 was added in defined proportions. 

**Figure 1:**
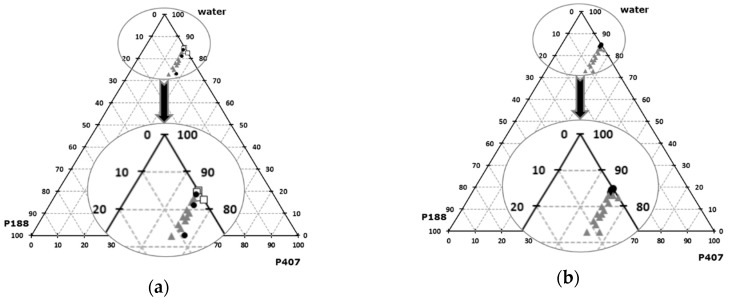
Ternary mix of Poloxamers 407/188 / H2O, sol-gel transition temperature (**a**), osmolarity (**b**). (**a**): 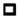 low T°sol-gel transition, 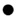 acceptable T°sol-gel transition, 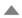 high T°sol-gel transition; (**b**): 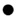 acceptable osmolarity, 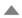 High osmolarity.

Various ternary mixtures of P407, P188, and water were tested. The gelation temperatures and osmolarity of theses formulations were plotted on ternary diagrams (Figure 1). For all the formulations, pH is acceptable (optimum pH for nasal rote is 6 ± 3). Increase of P407 relative content leads to a gelation temperature decrease, and to higher osmolarity values. P188 relative content increases both the gelation temperature and the osmolarity, as previously reported in the literature [62].

**Figure 2:**
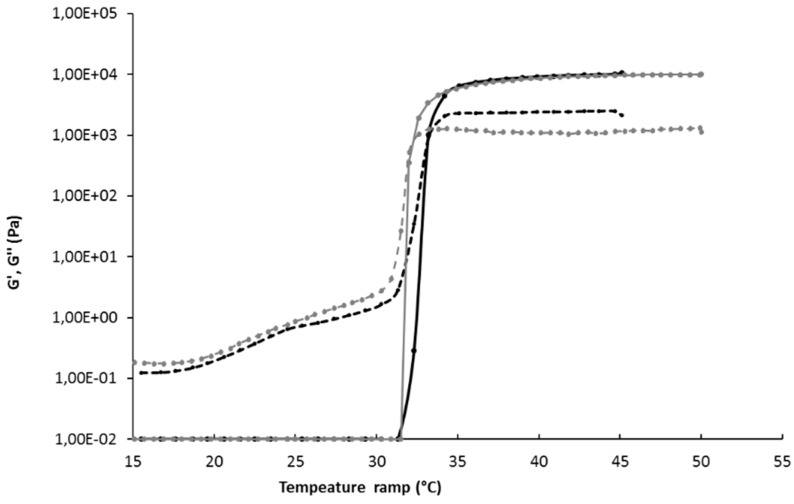
Rheology determination of gelation temperature of gel 15/1 with and without liposomes. Black continuous line G’ storage modulus of Gel 15/1, black broken line G’’ loss modulus of gel 15/1, grey continuous line G’ storage modulus of Gel 15/1 with liposome, grey broken line G’’ loss modulus of gel15/1 with liposome.

By using 15 wt% of P407 and 1 wt% of P188, an optimal formulation is obtained, characterized by a gelation temperature of 34.5 ± 0.3 °C, an osmolarity of 277 ± 4 mOsm, and a pH of 6.5. Gelation temperature of this P407/P188 mixture was confirmed by rheological determination, illustrated by the sudden increase of the storage modulus as the gel forms (Figure 2). When the storage modulus value overcomes the loss modulus value, the elastic behavior prevails on the viscous behavior. The physicochemical properties of this formulation are therefore suitable for intranasal administration according to the European pharmacopeia specifications [28], since the use of this isotonic formulation, with a stabilized pH value, would not adversely affect the functions of the nasal mucosa and its cilia.

### 3.4. Preparation of API Delivery Composite Formulation

In situ forming gel containing liposomes was prepared by the addition of poloxamers to an aqueous solution containing formulated liposomes. In these conditions, liposomes present higher mean hydrodynamic diameter values (140.8 ± 0.5 nm in the poloxamer solution at 25 °C vs. 119.0 ± 0.7 nm just after liposome formulation). The PDI remained lower than 0.1, and the zeta potential values remained broadly neutral (Table 2). This increase in the average liposome diameter included in the P407/P188 gel formulation may suggest an interaction between the poloxamers and the phospholipids forming the liposomes.

The addition of liposomes in gel formulation did not significantly modify the gelation temperature (Figure 2), nor the osmolarity (Table 3). The pH value decreased slightly but remained in the optimum value range. Free API also did not interfere with gel properties (Table 3). Thus, neither API nor liposome addition to P407/P188 (15/1 wt%) gel significantly changed the gel formulation properties that respect the nasal physiologic conditions.

**Figure 3:**
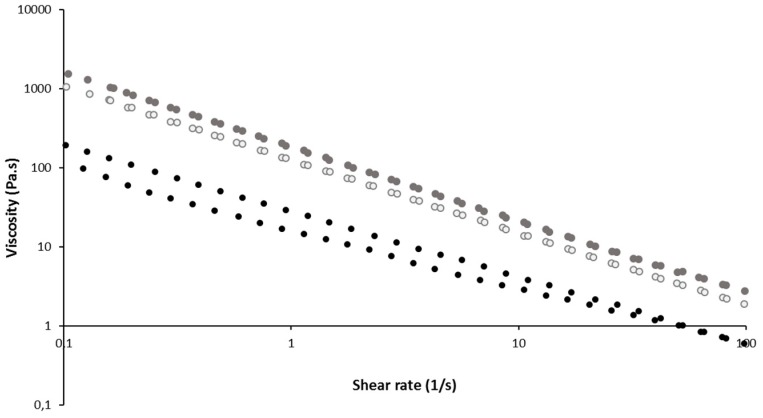
Shear stress-viscosity vs. deformation at 34 °C of gel 15/1 with and without liposomes (Shear-viscosity results). Black cercle: Gel 15/1, grey empty cercle: Gel 15/1 with liposome 60 mM, grey full cercle: gel 15/1 with liposome 120 mM.

The rheological behavior of gel formulations containing liposomes or not were determined during shear rate ramps. In all cases, the shear viscosity progressively decreased (shear thinning) when the shear rate increased. All formulations behaved as a shear-thinning (non-Newtonian) fluids. The presence of liposomes increased the viscosity regardless of the shear rate, and higher content of liposomes led to higher viscosity (Figure 3). Such a viscosity increase with the dispersed phase concentration is classically observed for emulsions or suspensions.

**Figure 4:**
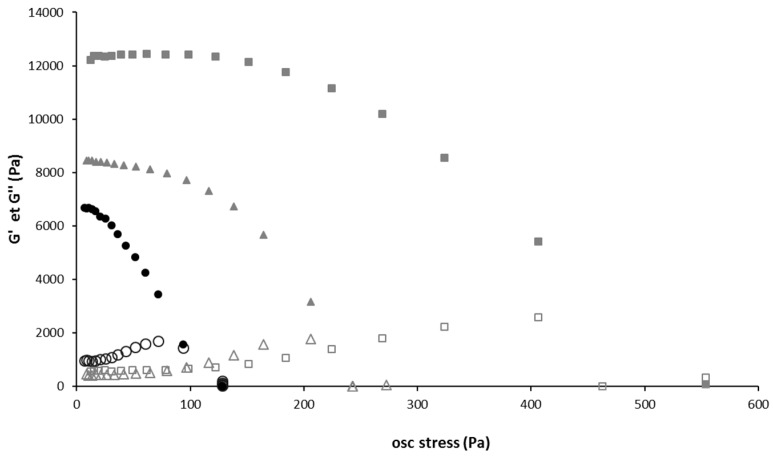
Strain sweep of gel with and without liposome.Storage (G’) and loss (G’’) moduli as a function of the oscillation stress. Black full circle: G’ storage modulus of Gel 15/1, grey full triangle: G’ storage modulus of Gel 15/1 with liposome 60 mM, grey full square: G’ storage modulus of Gel 15/1 with liposome 120 mM, Black empty circle: G’’ loss modulus of Gel 15/1, grey empty triangle: G’’ loss modulus of Gel 15/1 with liposome 60 mM, grey empty square: G’’ loss modulus of Gel 15/1 with liposome 120 mM.

To evaluate the strength of the gels above T_gel_, the evolution of storage (G′) and loss (G″) moduli of the gel and liposomes-containing gels were measured as a function of the oscillatory stress (Figure 4). For all gels, G′ was much higher than G″ at low oscillatory stress, which is typical of solid-like materials. The presence of liposomes in the hydrogel significantly increased the storage modulus, and this increase was higher if liposome concentration increased. The stress at which the hydrogel lost 50% of its stiffness also increased significantly with the liposome concentration. Thus, it appears that liposomes simultaneously increase the hydrogel’s stiffness and its strength. This leads us to think that liposomes interact with poloxamers, and play a role in the gel structuration. Moreover, the increase of liposomes average diameter in presence of poloxamers could also be related to interactions between poloxamers with the liposomes surface. Indeed, poloxamers exhibit surfactant properties and could interact with the phospholipid bilayer. Bochot et al. reported a destabilization of liposomes by P407 at low concentration [63]. P407 has a different behavior depending on its concentration in the formulation. Within dilute solutions (2%), its chains possess a greater fluidity that could lead to the insertion into phospholipid bilayer. A study of Wu and Lee also reports the existence of a saturation concentration of poloxamers, below which poloxamers can insert themselves in a lipid bilayer without disrupting liposomes and above which they instead disintegrate liposomes [64]. Polymer-liposomes complexes were prepared by Cho et al. The size of these complexes increased as the polymer content increased [65]. Polymer interactions with membranes would be due in particular to the central POP hydrophobic part of the copolymers [66]. There are two possibilities to explain the increase of liposome size: either the inclusion (association) of the polymers within the lipid bilayers or their adsorption on the vesicle surfaces. Based on adsorption method, Tian et al. prepared liposomes surfaces modified with P188 [67]. On the same way, the size of the liposomes gradually increased with increasing P188 content. Anyway, in our present study, whatever the precise interactions between poloxamers and liposomes, they do not affect sol-gel transition temperature but modify gel properties.

### 3.5. Mucoadhesion Properties

The thermosensitive properties of P407 have been widely exploited in the development of in situ nasal forming gels [68]. In contrast to P188, P407 is rather described as having limited mucoadhesion properties [26]. For this reason, it appeared interesting to integrate in the formulation a highly mucoadhesive polymer in order to optimize the interactions between the gel and the nasal mucosa. Chitosan and hydroxypropylmethyl cellulose (HPMC) are very widely described as mucoadhesive excipients, able to be incorporated in a gel in order to increase its mucoadhesion properties, and promote the API diffusion to the CNS [62]. 

In our studies, it appeared that the addition of excipients (HPMC or chitosan), at the defined classically used concentration of 0.5 wt%, didn’t significantly affect the sol-gel temperature (data not shown). 

**Figure 5:**
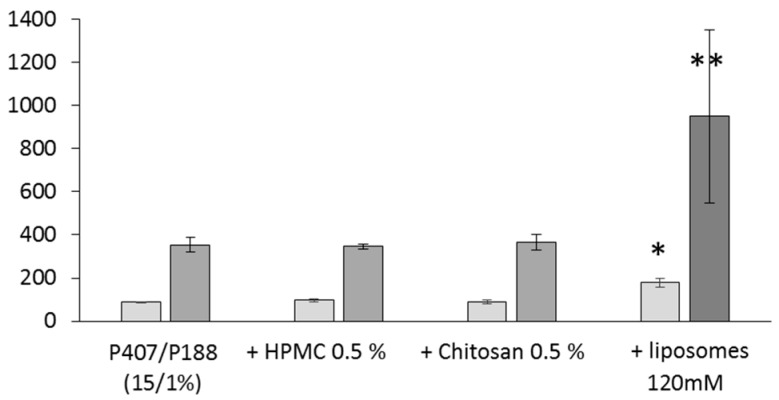
Mucoadhesion studies. Each point represents mean ± standard deviation (*n* = 4); *versus 15/1% *p* < 0.01; **versus 15/1% *p* < 0.001. 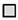 detachment Force (mN), 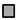 adhesion work (mN.mm).

Mucoadhesion performance of these formulations were assessed on a mucin disc (Figure 5). In the literature, when the concentration of HPMC increased from 0.1% to 1.0% in P407/P188/HPMC gel, an increase in mucoadhesive strength was measured from 4560 dyn/cm^2^ to 1 0935 dyn/cm^2^ [23]. Surprisingly, in our case, no difference was observed between mucoadhesion properties of the gel formulated either without or with 0.5 wt% HPMC or chitosan. For chitosan, this unexpected result could be due to the low molecular weight of the polysaccharide, that we used. Indeed, after comparing different types of chitosans, some authors previously showed that the increase in the chitosan molecular weight and viscosity could expand the adhesiveness of gels [69]. 

On the contrary, liposome addition in the gel led to a significant increase of the detachment force and of the adhesion work (Figure 5). Poloxamers are able to form entanglements or non-covalent bonds with mucus, thus favoring a greater degree of interaction with various biological tissues [21]. In our formulation, interactions between liposomes and poloxamers could impact the strength of the gel and further, positively influence their interactions with the mucin. In these conditions, an increased nasal residence time should be obtained, and so in better API bioavailability in the olfactory mucosa. It can be noted that the granulometric properties of the liposomes were kept unchanged at the end of these experiments (data not shown).

### 3.6. API In Vitro Release

**Figure 6:**
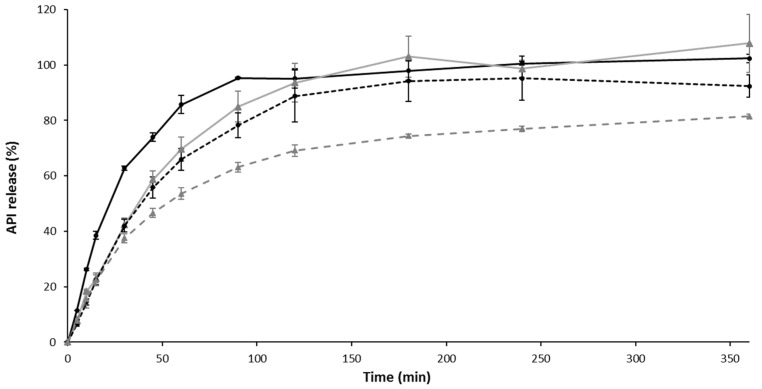
Active pharmaceutical ingredient (API) release in simulated nasal fluid. Each point represents mean ± standard deviation (n = 3). 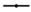 Solution API 1 mg/mL, 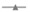 API-loaded liposomes , 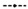 Gel with API 1mg/mL, 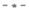 API-loaded Liposomes in gel.

Drug release from liposomes, hydrogels and composite formulation was performed in nasal simulated fluid, on Franz cells (Figure 6). 

A sustained API release was observed with liposomes or hydrogel, and a maximal delayed release was obtained with the composite formulation (the time to reach a 50% release of API, T_50_% = 60 min) in comparison with a drug solution (T_50_% = 20 min). Thus, it appears that, by modulating the nature of galenic form, and/or by using liposomes, the API diffuses according to different release kinetics. 

Already, in the literature, it was showed that liposome incorporation into a hydrogel promoted prolonged API release, resulting in increased API residence time at the site of administration. For example, calcein release from liposomal gels was found slower in comparison to control gels, and could be further retarded by using rigid-membrane liposomes, i.e., cholesterol-based liposomes as in our formulation [70]. It is known that drug release from liposomes mainly depends on lipid composition, the size of liposomes, and the extent of lipid packing [71]. 

To date, the ideal pharmacokinetics profile to reach is not defined for our studied API. Considering all our results, it will be possible to optimize it by using either the drug solution, the API hydrogel, the liposomal or the API composite formulation after nasal administration. 

The study of Al Asmari et al. showed that a more rapid and a greater transport of liposomal donepezil into the brain could be obtained, with similar release kinetic [12]. This probably would not be the case with our formulations. In the study of Arumugam et al., a significantly higher level of drug in the brain was found after intranasal administration of liposomes of rivastigmine compared to the intranasal administration of drug solution [14]. Moreover, these authors demonstrated the longer half-life in the brain after intranasal administration of liposomes than intranasal or oral administration of rivastigmine solution. In these conditions, the efficacy of the API into the CNS can be optimized. The best formulation to use will be defined from further in vivo pharmacokinetics and pharmacodynamics studies. 

## 4. Conclusions

We based our present study on an API, acting as a promising selective BuChE inhibitor. An innovative composite formulation was designed to enable administration of this API by intranasal route, thus allowing prolonged residence time in the nasal cavity, and a controlled drug release. Such a formulation strategy should permit not only to circumvent the low API BBB-permeability, but also to decrease its systemic exposure and its potential correlated cardiac toxicity effects, and also ultimately, to increase its bioavailability in the CNS.

This formulation consists in a thermosensitive gel, endowed with mucoadhesive properties and carrying liposomes of a promising API for the treatment of AD. The entire formulation is indicated and compatible for intranasal administration, a particularly advantageous route for the outpatient management of patients suffering from this major neurodegenerative disease and having no curative treatment to date. This composite formulation can be used as a whole, or each element (solution, thermosensitive gel, liposomes) can be taken separately, enabling a defined but different API release profile. Further in vivo experiments will contribute to determine ideal in vivo pharmacokinetics and pharmacodynamics of this API. The formulations that we developed will thus permit to optimize API delivery into the CNS in order to obtain a therapeutic effect in AD.

## Figures and Tables

**Table 1:** Human **Acetylcholinesterase** ((h) AChE) and equine **Butyrylcholinesterase** ((eq) BuChE) inhibitory activities (IC50) by Ellman method.

	(h) AChE IC50 (nM	(eq) BuChE IC50 (nM)
Donepezil	11 ± 3	-
Tacrine	-	2 ± 1
API	14,520 ± 1,485	573 ± 40

Note: values are expressed as mean ± standard error of the mean (SEM) of at least two experiments.

**Table 2:** Properties of the developed liposomes.

	Hydrodynamic Diameter (nm)	PDI	ZP (mV)	%EE	%DL	Concentration (mg/mL)
Blank liposomes	119.0 ± 0.7	0.032 ± 0.004	−5.5 ± 0.3	-	-	-
API-Lip	114.9 ± 2.4	0.048 ± 0.020	−11.2 ± 1.4	11.1 ± 1.0	1.4 ± 0.1	1.2 ± 0.1
Blank liposomes in gel	140.8 ± 0.5	0.078 ± 0.004	−6.6 ± 0.5	-	-	-

Note: values are expressed as mean ± standard deviation (*n* = 3), PDI: Poly Dispersity Index, ZP: Zeta potential, EE: The encapsulation efficiency, DL: drug loading.

**Table 3:** Properties of gels.

	Optimum	P407/P188 (15/1 wt %)	+API 1 mg/mL	+Liposomes 60 mM	+Liposomes 60 mM + API
Tsol-gel (°C)	32–35	34.5 ± 0.3	34.8 ± 1.2	30.9 ± 1.0	32.6 ± 1.0
Osmolarity (mOsm)	280 ± 20	277 ± 4	287 ± 6	280 ± 5	293 ± 5
pH	6 ± 3	6.5	6.5	5.5	5.5

Note: values are expressed as mean ± standard deviation (*n* = 3).
